# From Pathophysiology to Treatment: Contemporary Approaches to CFRD in the Pediatric and Adolescent Population

**DOI:** 10.1155/pedi/5539725

**Published:** 2026-01-16

**Authors:** Dogus Vuralli

**Affiliations:** ^1^ The Genetics and Genomic Medicine Research and Teaching Department, University College London (UCL) Great Ormond Street Institute of Child Health, 30 Guilford Street, London, WC1N 1EH, UK, ucl.ac.uk; ^2^ Department of Paediatric Endocrinology, Great Ormond Street Hospital for Children, London, UK, nhs.uk; ^3^ Department of Pediatric Endocrinology, Hacettepe University Faculty of Medicine, Ankara, Türkiye, hacettepe.edu.tr

**Keywords:** *CFTR* modulator therapy, cystic fibrosis, cystic fibrosis related diabetes, diabetes, glucose intolerance, glucose metabolism, oral glucose tolerance test (OGTT), treatment

## Abstract

Cystic fibrosis‐related diabetes (CFRD) is the most prevalent nonrespiratory complication of cystic fibrosis (CF), with its prominence growing as survival rates improve due to advances in CFTR modulator therapies. Its prevalence increases with age, affecting nearly 50% of patients with CF (PwCF) over 30 years old. CFRD primarily results from progressive pancreatic fibrosis leading to insulin deficiency, further compounded by intermittent insulin resistance during pulmonary exacerbations and systemic inflammation. Key risk factors include pancreatic insufficiency, female sex, severe *CFTR* genotypes (such as p.F508del homozygosity), CF‐related liver disease, and family history of type 2 diabetes. The early stages of CFRD are often asymptomatic, necessitating proactive screening. Annual oral glucose tolerance tests (OGTT) beginning at age 10 are challenging to perform but remain the gold standard for early detection, while continuous glucose monitoring (CGM) is increasingly recognized as a valuable complementary tool. Diagnosis is based on standard OGTT criteria, with indeterminate glycemia (INDET) and impaired glucose tolerance (IGT) recognized as prediabetic stages requiring close monitoring. Even early abnormalities in glucose metabolism may be associated with declines in pulmonary function and nutritional status, underscoring the need for rigorous surveillance and timely therapeutic intervention. Early initiation of insulin treatment can substantially mitigate these complications and improve clinical outcomes. Insulin remains the cornerstone of CFRD management, is recommended as the primary treatment for patients with CFRD (PwCFRD) rather than dietary modification alone. While pilot studies and observational cohorts have suggested potential benefits of early insulin treatment in individuals with early glycemic abnormalities such as INDET or IGT, findings from randomized controlled trials do not provide evidence to justify initiating insulin before CFRD is established. Management strategies should be individualized, with personalized glycemic targets. Insulin dosing aims to achieve the maximum tolerable dose to maintain a low HbA1c, control postprandial hyperglycemia without inducing hypoglycemia, minimize catabolism, and preserve optimal nutrition and pulmonary health without restricting carbohydrate intake. Regular glucose monitoring, quarterly HbA1c measurements, and annual screening for microvascular complications starting 5 years after diagnosis are essential to optimize outcomes. The advent of CFTR modulator therapies has revolutionized CF care, significantly improving outcomes and quality of life for PwCF. These therapies also show promise in improving glucose regulation and may impact the prevalence, onset, and course of CFRD. However, current data remain inconclusive, and the long‐term effects are still being elucidated. Future directions in CFRD research include refining screening protocols, exploring adjunctive noninsulin therapies, and developing predictive biomarkers. This review summarizes the current understanding of CFRD pathophysiology, diagnosis, and management strategies, emphasizing the importance of early intervention and personalized care in the context of evolving CF treatment approaches and their potential to improve prognosis for PwCF.

## 1. Introduction

Cystic fibrosis‐related diabetes (CFRD) is a well‐established consequence of cystic fibrosis (CF), commonly diagnosed in children over 10 years old [1]. Although distinct in its pathophysiology, CFRD shares certain characteristics with both type 1 (T1D) and type 2 diabetes (T2D). The primary mechanism underlying CFRD is relative insulin deficiency due to pancreatic islet destruction, with insulin resistance emerging during acute exacerbations and chronic pulmonary disease. Early CFRD is usually asymptomatic, with normal fasting blood glucose levels (BGLs), limiting the usefulness of fasting‐based screening. BGLs may fluctuate throughout the disease course, influenced by the patient’s clinical status and therapeutic interventions, often deteriorating with age. CFRD is commonly linked to declining pulmonary function, poor nutritional status, and an increased risk of pulmonary infections, and insulin treatment has been shown to improve both pulmonary and nutritional outcomes [2, 3]. Long‐term data indicate a six‐fold reduction in survival among patients with CFRD (PwCFRD) compared to patients with CF (PwCF) not having diabetes, with several studies reporting an even higher risk in females [4]. Close monitoring of BGLs and early initiation of insulin treatment are crucial for effective CFRD management, helping to mitigate complications and improve patient outcomes.

## 2. Epidemiology

### 2.1. Prevalence of CFRD

CFRD prevalence increases substantially with age. Data from the Minnesota CF Center—where annual oral glucose tolerance test (OGTT) screening has been conducted in patients aged ≥6 years since the early 1990s—reported rates of 2% in individuals ≤10 years, 19% in those aged 11–17, 40% in adults aged 18–29, and 45%–50% in those ≥30 years [5]. Similarly, the 2020 CF Foundation registry reported CFRD in 10.5% of patients under the age of 19 and 56.8% of those ≥20 years [6]. German and Austrian registry data indicate prevalence rates of 11% at age 20 and 25% at age 35 [7]. Recent studies also show a rise in diagnosed CFRD cases—from 2.0 to 22.1 per 1000 person‐years—likely reflecting improved screening and increased life expectancy among PwCF rather than a true increase in incidence [8].

### 2.2. Risk Factors for CFRD

CFRD develops as a consequence of progressive pancreatic damage; thus, the most significant risk factors are indicators of pancreatic disease. Major risk factors include exocrine pancreatic insufficiency, genotypes associated with severe disease, advanced age, female sex, CF‐related liver disease, and a family history of T2D. PwCF with preserved pancreatic function have a lower likelihood of progressing to CFRD relative to those with pancreatic insufficiency, though their risk remains substantially higher than that of the general population [9]. CFRD is particularly common in individuals with severe CFTR (CF transmembrane conductance regulator) variants—especially p.Phe508del homozygosity—which are strongly associated with pancreatic insufficiency. Age is a major determinant of CFRD risk, with prevalence increasing substantially over time. CFRD is more frequent in females, especially after the age of 30, who also experience reduced pulmonary function and lower survival rates than males, contributing to a shorter life expectancy [4, 5]. The presence of CFRD has been linked to greater declines in pulmonary function—reflected by lower forced expiratory volume in 1 s (FEV_1_), and more frequent pulmonary exacerbations—and this deterioration often precedes CFRD diagnosis but improves after insulin treatment, suggesting that the impairment in pulmonary function is a consequence of CFRD rather than its underlying cause [10, 11]. Nutritional status is another critical factor influencing CFRD risk, as patients with poor nutritional status are more likely to develop CFRD, although this association may also reflect the impact of severe CF genotypes, pancreatic insufficiency, and other CF‐related complications. CF‐related liver disease is similarly associated with increased CFRD risk, potentially through its overlap with these factors, although a direct effect of liver disease on glucose metabolism cannot be excluded.

Emerging evidence indicates that genetic predisposition contributes to CFRD risk. Individuals with a familial predisposition to T2D or who carry variants in T2D‐associated genes—such as *TCF7L2*, *CDKAL1*, *CDKN2A/B*, and *IGF2BP2*—have an increased risk of developing CFRD [12–14]. Specifically, variants in the *TCF7L2* gene—known to influence *β*‐cell growth and insulin secretion and recognized as a susceptibility factor for T2D—increase CFRD risk in PwCF by approximately threefold [12, 15, 16]. Further evidence for shared genetic factors between CFRD and T2D comes from the higher concordance of CFRD in monozygotic compared to dizygotic twins. Moreover, variants in the *SLC26A9* gene, which produces an anion transporter expressed in parallel with CFTR in pancreatic ductal cells, have been linked to earlier CFRD onset, but not to T2D [13, 14]. Variants in inflammatory genes such as *TNF* and *Calpain-10* may also contribute to *β*‐cell dysfunction and impaired glucose metabolism in CFRD [17, 18].

**Key Practice Points—Risk Factors for CFRD:**

• The strongest risk factors for CFRD are markers of pancreatic damage, including exocrine pancreatic insufficiency and severe *CFTR* genotypes (especially p.Phe508del homozygosity).
• Age is a major determinant, with CFRD prevalence rising steadily over time; females—particularly after age 30—have higher risk and worse outcomes.
• Poor nutritional status, CF‐related liver disease, and more severe CF phenotypes increase CFRD risk, though these factors often overlap with pancreatic insufficiency and genotype severity.
• Genetic susceptibility plays a role: family history of T2D and variants in T2D‐associated genes (e.g., *TCF7L2*, *CDKAL1*, *CDKN2A/B*, *IGF2BP2*) increase CFRD risk.
• Variants in *SLC26A9*, involved in pancreatic ductal ion transport, are associated with earlier CFRD onset, suggesting CF‐specific genetic modifiers.
• Additional contributors include inflammatory gene variants (e.g., *TNF*, *Calpain-10*) that may impair *β*‐cell function and glucose metabolism.

## 3. Pathophysiology

CFRD primarily results from progressive insulin deficiency due to gradual loss of pancreatic tissue. While the precise mechanisms underlying insulin insufficiency remain incompletely understood, contributing factors include exocrine pancreatic damage, chronic inflammation, genetic predisposition, and nutritional status. Dysfunction of the chloride channel leads to the production of thick, viscous secretions that obstruct the exocrine pancreas, resulting in progressive fibrosis, fat accumulation, and destruction of pancreatic islets, ultimately depleting *α*, *β*, and pancreatic polypeptide‐secreting cells [19]. The development of CFRD is primarily attributed to *β*‐cell loss and inflammation within the islets, rather than intrinsic islet dysfunction, and interleukin‐1*β* immunoreactivity within the islets has been implicated in the early stages of this destructive process [20, 21].

Insulin serves a crucial role in maintaining airway glucose homeostasis, a process that is impaired in CF [22]. PwCFRD have higher airway glucose concentrations than those with CF alone, promoting bacterial proliferation—particularly *Staphylococcus aureus* and *Pseudomonas aeruginosa*—and exacerbating inflammation, which may contribute to the pulmonary decline associated with CFRD [23]. Despite increased neutrophil presence in respiratory secretions, hyperglycemia has been shown in murine models to interfere with bacterial clearance [24]. It is also linked to increased lactate secretion onto airway surfaces, contributing to acidification of the airway surface fluid, which disrupts ion transport, impairs epithelial repair, triggers inflammation, and affects voltage‐dependent potassium channels essential for mucus clearance [25–27].

CFRD progresses through two clinical phases; in early disease, postprandial hyperglycemia predominates and can be detected by continuous glucose monitoring (CGM) or 2‐h OGTT with 30‐min sampling, revealing transient postprandial glucose fluctuations indicative of an impaired first‐phase insulin response. This early‐phase insulin deficiency is often accompanied by mild insulin resistance [28–30]. Unlike T1D, CFRD is also associated with impaired glucagon secretion, reflecting widespread islet destruction [31]. Insulin resistance becomes more prominent in the later stages of CFRD and during acute pulmonary exacerbations, though it may be present to a lesser extent in the early phases of the disease [32]. During acute exacerbations, increases in stress hormones—growth hormone, cortisol, and catecholamines—together with inflammatory cytokines further worsen insulin resistance, helping explain why CFRD is more common in older PwCF than in younger individuals [33].

Fasting hyperglycemia (FH) typically emerges in the advanced stages of CFRD, when hemoglobin A1c (HbA1c) levels may still be normal or only begin to rise [34]. Classical diabetic symptoms usually develop during acute pulmonary exacerbations or in older individuals, reflecting the concurrent worsening of insulin deficiency and insulin resistance [33, 34]. Prolonged hyperglycemia can further aggravate insulin resistance by reducing GLUT4 expression [35, 36]. While postprandial hyperglycemia is recognized as the earliest and most sensitive indicator of CFRD, FH emerges as a later marker.

**Figure 1:**
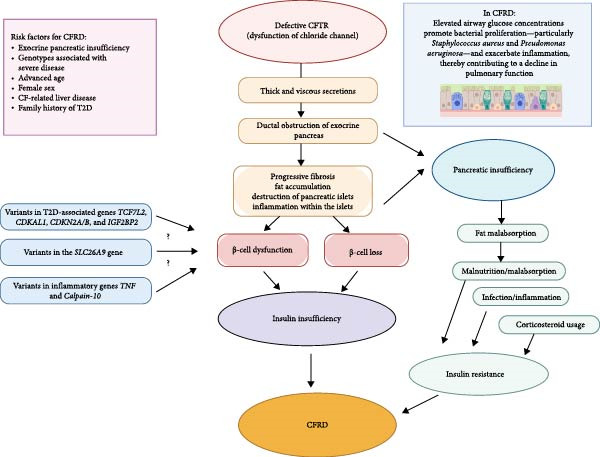
Pathophysiology of cystic fibrosis related diabetes. CFRD, cystic fibrosis related diabetes; CFTR, cystic fibrosis transmembrane conductance regulator; T2D, type 2 diabetes.

A variety of CF‐associated conditions can further disrupt glucose metabolism, including malnutrition, malabsorption, acute and chronic infections, glucagon deficiency, liver dysfunction, increased respiratory effort, and high energy expenditure. Additionally, corticosteroids used for allergic bronchopulmonary aspergillosis and immunosuppressive agents such as tacrolimus after lung transplantation significantly contribute to glucose intolerance and increase the risk of CFRD. Factors involved in the pathophysiology of CFRD are shown in Figure 1.

**Key Practice Points—Pathophysiology of CFRD:**

• CFRD arises from progressive insulin deficiency due to exocrine pancreatic destruction and islet inflammation.
• Hyperglycemia increases airway glucose and acidification, promoting infection, inflammation, and pulmonary decline.
• Early disease shows postprandial hyperglycemia from impaired first‐phase insulin secretion; glucagon deficiency and later‐stage insulin resistance further worsen glycemic control.
• Fasting hyperglycemia appears in advanced disease, and chronic hyperglycemia amplifies insulin resistance.
• Malnutrition, infections, liver dysfunction, steroids, and tacrolimus further impair glucose metabolism and increase CFRD risk.

## 4. Clinical Presentation

The clinical presentation of CFRD varies widely depending on the severity of underlying CF pathology and the stage of the disease, and its onset is often insidious. Early in the course, CFRD may be asymptomatic and identified only through routine screening. Progression is typically gradual, with most patients experiencing clinical decline—such as unexplained, chronic reductions in pulmonary function and deteriorating nutritional status—before classic diabetic symptoms appear. Subclinical hyperglycemia may further worsen pulmonary disease by promoting infection and inflammation, leading to more frequent pulmonary exacerbations, declines in FEV_1_, and increased hospitalization rates. Malnutrition and inadequate weight gain can be the hallmark indicators of CFRD, particularly in children and adolescents, and nutritional deterioration may precede detectable hyperglycemia, emphasizing the importance of proactive surveillance. CFRD may first manifest during periods of increased insulin resistance, such as during acute pulmonary exacerbations, corticosteroid administration, or periods of increased carbohydrate intake—particularly during continuous nocturnal enteral tube feeding. Overt CFRD may present with classic symptoms of hyperglycemia, including excessive thirst, increased urination, unintentional loss of body mass or difficulty achieving expected weight gain even with adequate caloric intake, and poor growth velocity. Unlike T1D, diabetic ketoacidosis is rare in CFRD because residual endogenous insulin secretion is typically preserved; its occurrence should prompt evaluation for possible coexisting T1D.

### 4.1. Clinical Impact on Pulmonary Function

Numerous studies have demonstrated the detrimental effects of insulin deficiency and glucose metabolism abnormalities on pulmonary outcomes in PwCFRD. In a cohort of 377 pediatric PwCF older than 7 years, pulmonary function declined more rapidly in those with CFRD than in those without diabetes [37]. Evidence suggests that declines in pulmonary function and nutritional status may begin several years before CFRD is formally diagnosed [38]. Moreover, defects in insulin secretion, as assessed by OGTT, have been correlated with early pulmonary impairment, demonstrated by ventilation inhomogeneity on the multiple breath washout test [39]. Similarly, in a study assessing 24 PwCFRD without FH, 59 with impaired glucose tolerance (IGT), and 69 with normal glucose tolerance (NGT), both FEV_1_ and forced vital capacity (FVC) were reduced across all groups, with the greatest decline observed in PwCFRD without FH [10]. Furthermore, patients with the lowest baseline insulin secretion showed the most pronounced decline in pulmonary function over a 4‐year follow‐up, suggesting a strong association between insulin deficiency and worsening clinical outcomes [10].

### 4.2. Clinical Impact on Nutritional Status

A strong association exists between CFRD and nutritional status, with declines in nutritional indices often preceding diagnosis. Insulin is a potent anabolic hormone essential for maintaining lean body mass; its deficiency induces a catabolic state which, together with exocrine pancreatic insufficiency, malabsorption, and increased energy expenditure due to respiratory effort, contributes to the poor nutritional outcomes in PwCFRD. In a longitudinal study involving 61 PwCFRD without FH, body mass index (BMI) declined during the year preceding insulin initiation but increased by 0.39 ± 0.21 kg/m^2^ following treatment [40]. Even mild glucose abnormalities, such as IGT, have been linked with nutritional deterioration and weight loss before the onset of overt CFRD [41]. Additionally, among PwCF with NGT, those with the lowest BMI showed greater declines in insulin secretion, suggesting that progressive insulin deficiency contributes to worsening nutritional status even before CFRD develops [42]. Collectively, these findings suggest that insulin deficiency contributes to BMI decline, and early intervention with insulin may have favorable effects prior to the overt manifestation of CFRD.

More recent evidence, however, suggests that the anabolic response to insulin may be limited in contemporary, clinically stable PwCF. In a double‐blind, placebo‐controlled randomized clinical trial (RCT) by Schiavon et al. [43], 30 participants aged 10–25 years with abnormal glucose tolerance (IGT or indeterminate glycemia [INDET]) but without CFRD—17 of whom (57%) were on CFTR modulators—were randomized to receive once‐daily long‐acting insulin, premeal rapid‐acting insulin, or placebo for 4 weeks. Contrary to earlier reports of increased protein breakdown in CF, baseline protein catabolism was comparable to that of healthy controls, and no significant differences in any indices of protein turnover—including endogenous protein breakdown—were observed between insulin‐ and placebo‐treated groups, suggesting improved metabolic status in contemporary CF populations [43].

Overall, these findings suggest that although progressive insulin deficiency remains a major determinant of nutritional decline in CF, advances in clinical care and CFTR modulator therapies have substantially improved baseline nutritional and metabolic status, reflecting the evolving clinical landscape of modern CF management.

### 4.3. Micro and Macrovascular Complications

Advances in CF management and improved life expectancy have led to a higher recognition of microvascular complications in PwCFRD, with peripheral neuropathy and diabetic gastroenteropathy being the most frequently reported. In a large cohort microvascular complications were identified in nearly half of the PwCFRD, with prevalence rates comparable to those observed in other forms of diabetes [44]. Among patients with FH, 16% exhibited diabetic retinopathy, and 14% had nephropathy, although these rates were lower than those typically reported in T1D. Notably, microvascular complications in CFRD tend to arise at lower HbA1c levels than in other diabetes types [44]. These complications typically develop after a relatively short duration of diabetes—approximately 10 years—and are closely associated with suboptimal glycemic control. Consequently, regular evaluation for microvascular complications is advised to start 5 years following a CFRD diagnosis [44, 45].

Macrovascular complications are rare but remain a potential concern in CFRD [46]. Case reports have documented symptomatic myocardial infarction in PwCFRD, and postmortem studies have revealed myocardial fibrosis and necrosis [47, 48]. Preliminary vascular abnormalities have also been reported even in the absence of hypertension, suggesting an increased risk of cardiovascular complications. In a study evaluating arterial stiffness in 50 PwCF—13 with CFRD and 37 without diabetes—compared with 26 healthy controls, the augmentation index, a marker of arterial stiffness, was significantly higher in PwCFRD than in both nondiabetic PwCF and healthy controls [49]. These findings suggest that CFRD is associated with increased arterial stiffness, reflecting early vascular changes in this population.

**Key Practice Points—Clinical Presentation of CFRD:**

• CFRD often develops insidiously, with early stages frequently asymptomatic and detected only through routine screening.
• Declining pulmonary function and deteriorating nutritional status often precede overt hyperglycemia and may be the earliest clinical indicators.
• CFRD may first manifest during periods of increased insulin resistance (e.g., pulmonary exacerbations, corticosteroid treatment, nocturnal enteral feeding).
• Classic hyperglycemic symptoms (polydipsia, polyuria, weight loss) may occur with overt disease, while DKA is rare and should prompt evaluation for coexisting T1D.

**Key Practice Points—Impact on Pulmonary Function:**

• Insulin deficiency and glucose abnormalities are strongly linked to accelerated declines in FEV_1_ and FVC, often beginning years before diagnosis.
• Reduced insulin secretion correlates with worse pulmonary outcomes and predicts faster decline in pulmonary function.

**Key Practice Points—Impact on Nutritional Status:**

• Insulin deficiency contributes to catabolism and BMI decline, often preceding CFRD diagnosis.
• Insulin treatment improves BMI in PwCFRD, though metabolic responses may be less pronounced in modern CFTR–modulator–treated populations.
• Even mild dysglycemia (IGT/INDET) can negatively affect growth and nutritional status.

**Key Practice Points—Micro- and Macrovascular Complications:**

• PwCFRD can develop microvascular complications at lower HbA1c levels; screening should begin 5 years after diagnosis.
• Macrovascular complications are uncommon, but early vascular changes (e.g., increased arterial stiffness) have been observed.

### 4.4. Hypoglycemia in PwCF

**Figure 2:**
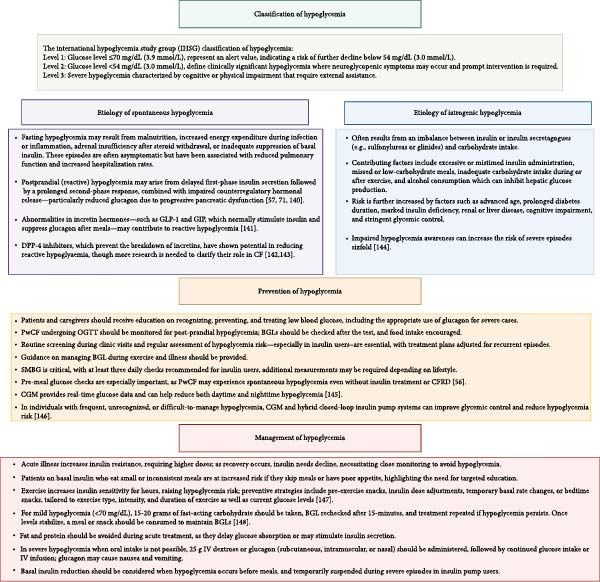
Classification, etiology, prevention, and management of hypoglycemia in patients with cystic fibrosis. BGL, blood glucose level; CF, cystic fibrosis; CFRD, cystic fibrosis related diabetes; CGM, continous glucose monitoring; DPP‐4, dipeptidyl peptidase‐4; IV, intravenous; GIP, glucose‐dependent insulinotropic polypeptide; GLP‐1, glucagon‐like peptide‐1; OGTT, oral glucose tolerance test; PwCF, patients with cystic fibrosis; SMBG, self‐monitoring of blood glucose.

**Figure 3:**
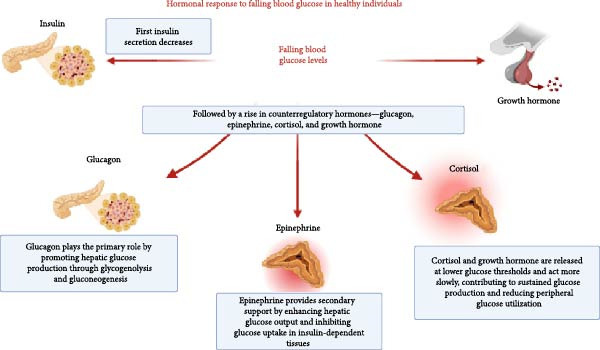
Hormonal responses to falling blood glucose levels in healthy individuals. Response to falling BGLs in T1D, T2D, and CF: In T1D and advanced T2D, the ability to counter hypoglycemia is impaired due to defective insulin regulation and a blunted glucagon response, leading to increased reliance on epinephrine to recover from hypoglycemia; recurrent hypoglycemia may further desensitize this response, resulting in hypoglycemia unawareness. In CF, glucagon secretion is similarly reduced—particularly in those with pancreatic insufficiency—likely reflecting CFTR‐related α‐cell dysfunction, while epinephrine responses are generally preserved. CF, cystic fibrosis; CFTR, cystic fibrosis transmembrane conductance regulator; T1D, type 1 diabetes; T2D, type 2 diabetes.

Hypoglycemia—defined as BGLs below 70 mg/dL (3.9 mmol/L) in individuals receiving glucose‐lowering treatments such as insulin—is an important concern in PwCF. Spontaneous hypoglycemia can also occur in CF, independent of CFRD or its treatment, and is typically defined as BGLs ≤47 mg/dL (2.6 mmol/L) [50]. The International Hypoglycemia Study Group (IHSG) established standardized glucose thresholds, later endorsed by the American Diabetes Association (ADA) and the European Association for the Study of Diabetes (EASD), to harmonize definitions and ensure consistent evaluation of hypoglycemia risk, emphasizing that such standardization is essential for identifying glucose levels associated with acute and chronic risks [51] (Figure 2). Symptoms of hypoglycemia range from neurogenic (e.g., tremors and palpitations) to neuroglycopenic (e.g., confusion and blurred vision), with severe cases potentially leading to seizures or coma. In healthy individuals, falling BGLs trigger a coordinated hormonal defense cascade, whereas in T1D, T2D, and CFRD important differences in the hormonal response are observed (Figure 3). Hypoglycemia and the fear of it can significantly reduce quality of life, affecting daily activities, and contributing to mood disorders like depression and anxiety [52, 53]. In CFRD, the severity of symptoms—particularly neuroglycopenic manifestations—has a greater impact on well‐being than episode frequency [54]. The etiology, prevention, and management of hypoglycemia in PwCF are summarized in Figure 2.

*Spontaneous Hypoglycemia*: Even without diabetes or glucose‐lowering treatments, PwCF may experience spontaneous hypoglycemia during fasting or postprandially. Reported prevalence varies widely (7%–69%) due to differences in definitions and study designs [55]. In one study of 129 PwCF undergoing OGTT, 14% had fasting BGL <60 mg/dL and 15% experienced reactive hypoglycemia (BGL <50 mg/dL), yet none reported symptoms even at very low glucose levels [56]. These episodes are typically asymptomatic, self‐limiting, and often detected during or after OGTT or CGM use, raising concerns about under‐recognition and possible hypoglycemia unawareness [57, 58]. Extended 3‐h OGTTs are more likely to detect reactive hypoglycemia than standard 2‐h tests, while CGM has proven to be more sensitive in detecting spontaneous hypoglycemia in daily life [55, 59, 60]. In a study of 45 children and adolescents with CF, 13.3% experienced hypoglycemia during a 2‐h OGTT, whereas 27.5% spent at least 3% of time in hypoglycemia over 3 days of CGM monitoring, with higher rates observed in females [61]. Hypoglycemia detected by CGM was more frequent than expected and exceeded that identified by OGTT, and all episodes were asymptomatic. These findings suggest that CGM may be more sensitive than OGTT for detecting asymptomatic or spontaneous hypoglycemia in PwCF; however, CGM can occasionally produce spurious low readings—for example, due to sensor compression during sleep—so low CGM values without corresponding symptoms should always be confirmed by fingerprick glucose measurements. Although reactive hypoglycemia during OGTT was once considered a marker of CFRD risk due to underlying insulin dysregulation, recent studies have found no such association, with some even suggesting a lower risk [62–64]. However, both reactive hypoglycemia and high glucose variability on CGM have been linked to reduced pulmonary function and lower FEV_1_ in PwCF, independent of nutritional status, underscoring the need for further research into the clinical impact of asymptomatic hypoglycemia [61].

*Iatrogenic hypoglycemia in CFRD*: Insulin treatment in CFRD improves nutritional status and pulmonary function but carries a risk of iatrogenic hypoglycemia. While severe episodes are uncommon, nonsevere hypoglycemia is frequently observed during insulin use and hospitalization. Studies indicate that 16%–58% of PwCFRD receiving insulin or other glucose‐lowering treatments experience nonsevere hypoglycemia, with some reporting multiple weekly episodes [40, 54]. In hospitalized patients, ~6.1% of glucose readings fall within the hypoglycemic range, particularly at night, although glucometer accuracy may be reduced in this setting [65].

*CFTR modulators effect on hypoglycemia*: CFTR modulators, particularly ivacaftor (IVA), have been shown to improve insulin secretion in PwCF—most notably in those carrying gating mutations such as G551D—suggesting potential restoration of islet function and improved glucose regulation [66, 67]. However, reports of hypoglycemia—especially in patients previously on insulin—suggest that modulators may also alter glucagon secretion, raising the risk of hypoglycemia in CFRD [68, 69]. While CFTR modulators have improved glycemic control in some individuals, they have also been associated with hypoglycemia, highlighting the need for close monitoring and further research to clarify these effects.

Significant gaps remain in understanding hypoglycemia in CF, including its prevalence, risk factors, underlying mechanisms, and impact on quality of life. Further research is needed to better define spontaneous hypoglycemia, determine its clinical relevance, and evaluate the role of CGM and closed‐loop systems in improving detection and management.

**Key Practice Points—Hypoglycemia in PwCF:**

• Hypoglycemia occurs in PwCF both with and without CFRD; spontaneous hypoglycemia is common, often asymptomatic, and may reflect altered counter‐regulatory responses.
• CGM detects more frequent hypoglycemia than OGTT or SMBG, but low CGM values should be confirmed by fingerstick testing due to possible sensor artifacts.
• Reactive or spontaneous hypoglycemia is not predictive of CFRD, though both hypoglycemia and high glucose variability have been linked to reduced pulmonary function.
• Iatrogenic hypoglycemia is common in insulin‐treated CFRD, though severe episodes are rare; careful insulin titration and monitoring are essential.
• CFTR modulators—especially ivacaftor—may increase hypoglycemia risk by altering insulin and glucagon secretion, particularly in PwCFRD previously on insulin.
• Hypoglycemia significantly affects quality of life, with neuroglycopenic symptoms having the greatest impact; ongoing patient education and individualized prevention strategies are required.
• Major knowledge gaps remain regarding the prevalence, mechanisms, and clinical significance of spontaneous hypoglycemia, highlighting the need for further research.

### 4.5. Mortality

While CFRD is associated with increased mortality, particularly among females, early detection and appropriate intervention can significantly mitigate this risk. In a comprehensive single‐center study of 872 PwCF, mortality among those with CFRD declined markedly across three consecutive 5‐year periods, paralleling overall improvements in CF survival [5]. Mortality rates in PwCFRD have decreased by nearly 50%, reaching 3.2 deaths per 100 patient‐years in females and 3.8 in males, effectively eliminating the gender disparity in CFRD‐related mortality. These findings underscore how crucial timely detection and appropriate care are in narrowing mortality gaps between genders and between PwCF with and without diabetes. However, despite significant improvements in survival, some reports indicate that PwCFRD over age 30 still experience higher mortality rates than age‐matched PwCF without diabetes [70]. Ongoing efforts to optimize screening, early intervention, and long‐term management remain essential to further reduce mortality in this population.

## 5. Screening and Diagnosis

Annual screening for CFRD is recommended for all PwCF starting at age 10, though some centers initiate screening as early as age 6 due to the early onset of IGT [71–75]. Several studies have shown that 42%–78% of children aged 6–9 exhibit IGT or INDET, both of which increase the risk of developing CFRD in early adolescence [75–77]. Emerging evidence also shows that IGT can develop as early as 3 months to 5 years of age, supporting consideration of screening in younger children [78]. The International Society for Pediatric and Adolescent Diabetes (ISPAD) supports early screening in young children, recognizing prediabetic states like IGT and INDET as predictors of CFRD progression, impacting nutritional status and pulmonary function [79]. Screening should typically be performed using an OGTT or CGM during clinically stable periods, as infections may induce transient insulin resistance. It is particularly indicated in cases of declining pulmonary function or worsening nutritional status suggestive of CFRD. Additional screening beyond annual assessments is recommended in specific conditions (Figure 4A). When to perform screening and the tests used for the screening and the diagnosis of CRFD are given in Figure 4.

Figure 4(A) When to perform screening (B) The tests used for the screening and the diagnosis of CFRD. ADA, American Diabetes Association; BGL, blood glucose level; CF, cystic fibrosis; CFRD, cystic fibrosis related diabetes; CGM, continuous glucose monitoring; INDET, indeterminate glycemia; IFG, impaired fasting glucose; IGT, impaired glucose tolerance; OGTT, oral glucose tolerance test; PwCF, patients with cystic fibrosis; PwCFRD, patients with cystic fibrosis related diabetes; T1D, type 1 diabetes; T2D, type 2 diabetes.

**Figure:**
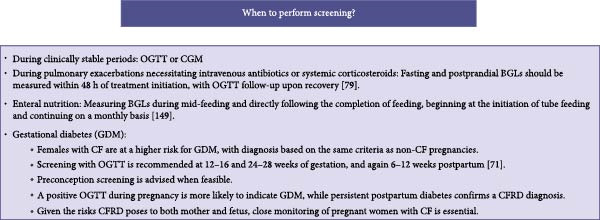
(A)

**Figure:**
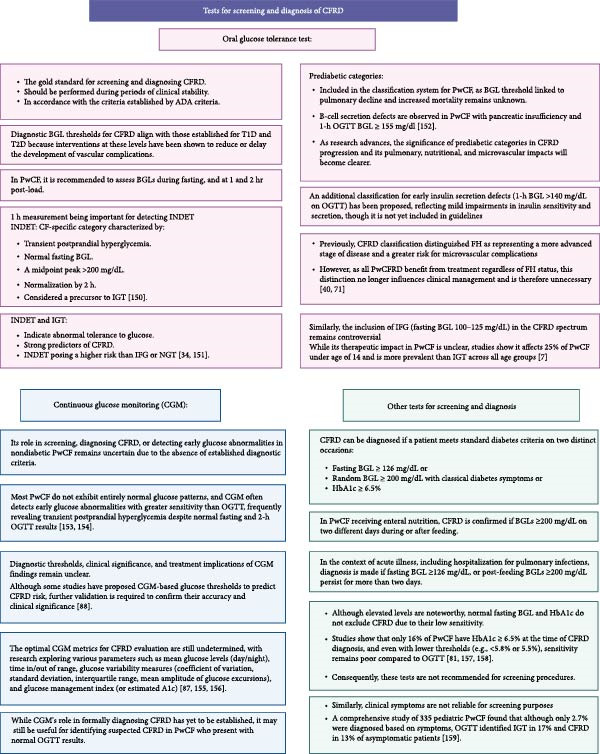
(B)

### 5.1. OGTT

The OGTT remains the gold standard approach for screening and diagnosing CFRD and should be performed during clinically stable periods in line with ADA criteria. The North American CFRD Guidelines Committee endorses OGTT over fasting BGL and HbA1c because of its superior accuracy, its association with nutritional and pulmonary deterioration, and its ability to facilitate early insulin treatment to prevent CF‐ and diabetes‐related complications [71]. Details of the tests used for screening and diagnosis are shown in Figure 4B, and the classification of abnormal glucose tolerance in PwCF is presented in Table 1. Previously, CFRD classification distinguished FH as representing a more advanced stage of disease and a greater risk for microvascular complications. However, as all PwCFRD benefit from treatment regardless of FH status, this distinction no longer influences clinical management and is therefore unnecessary [40, 71].

**Table 1:** Classification of abnormal glucose tolerance in patients with cystic fibrosis.

Name of the category	Fasting plasma glucose	Plasma 2‐h glucose at OGTT	Impact on clinical consequences
Normal glucose tolerance (NGT)	<126 mg/dL	<140 mg/dL	No adverse clinical impact
Indeterminate glycemia (INDET)	<126 mg/dL	<140 mg/dL, with a peak ≥200 mg/dL at 1‐h	Clinical trials signify a higher likelihood of morbidity
Impaired glucose tolerance (IGT)	<126 mg/dL	140–200 mg/dL	
CFRD without fasting hyperglycemia (FH)	<126 mg/dL	≥200 mg/dL	Increase in both morbidity and mortality rates
CFRD with fasting hyperglycemia (FH)	≥126 mg/dL	OGTT not necessary	
Impaired fasting glucose (IFG)	110–126 mg/dL	<140 mg/dL	

*Note*: Modified from: Ode KL, Ballman M, Battezzati A, Brennan A, Chan CL, Hameed S, Ismail HM, Kelly A, Moran AM, Rabasa‐Lhoret R, Saxby NA, Craig ME. ISPAD Clinical Practice Consensus Guidelines 2022: Management of cystic fibrosis‐related diabetes in children and adolescents. Pediatr Diabetes. 2022;23(8):1212−1228.

Abbreviations: CFRD, cystic fibrosis related diabetes; OGTT, oral glucose tolerance test.

The applicability of standard OGTT thresholds in CFRD diagnosis is also questioned, as they originate from non‐CF populations, and PwCF exhibit earlier and more pronounced postprandial hyperglycemia than non‐CF individuals. A study comparing nondiabetic PwCF with age‐ and BMI‐matched controls found that, despite similar HbA1c, fasting and 2‐h postload BGLs, PwCF exhibited higher postprandial BGLs at 30, 60, and 90 min [80]. Additionally, CGM data revealed higher mean glucose levels (106 vs. 92 mg/dL), and a greater proportion of glucose readings >200 mg/dL (33% vs. 5%) compared to controls, highlighting the potential need for CF‐specific diagnostic criteria.

An isolated abnormal OGTT does not necessarily require treatment but signals abnormal glucose tolerance (INDET or IGT), warranting close monitoring. This includes earlier repeat OGTT, BGL tracking during infections, and observation of nutritional or pulmonary decline, which often precedes CFRD onset. Progression from INDET to overt CFRD with FH is common, though variations in levels of severity are possible. OGTT results can fluctuate over time, as evidenced by two long‐term studies, with one reporting 22% worsening and 18% improvement in glucose tolerance, while another found 58% of patients with IGT later had normal OGTT results [10, 81]. These variations may be influenced by intervening medical conditions such as acute pulmonary infections affecting insulin sensitivity [82]. CFRD is diagnosed at the first instance of meeting criteria, regardless of any subsequent improvement in glucose tolerance.

OGTT‐based insulin measurements can detect early secretion defects linked to CFRD. Studies show nondiabetic PwCF have lower and delayed insulin peaks compared to healthy controls (120 min vs. 30–60 min) [83]. Although assessments of insulin secretion are not currently part of CFRD criteria, they may provide additional insights.

### 5.2. Other Tests for Screening and Diagnosis

While OGTT remains the primary diagnostic tool for CFRD, other tests may help in specific cases. The tests used for the screening and the diagnosis of CRFD are given in Figure 4B.

### 5.3. Role of CGM

CGM is widely used for tracking glycemic levels during insulin treatment, proving valuable for insulin dose adjustments and hypoglycemia detection in these patients. However, its role in screening, diagnosing CFRD, or detecting early glucose abnormalities in nondiabetic PwCF remains uncertain due to the absence of established diagnostic criteria (Figure 4B) [84, 85]. Recent studies link CGM‐detected glucose elevations to adverse outcomes such as weight loss, pulmonary function decline, and increased *Pseudomonas aeruginosa* colonization, even in PwCF with normal OGTT results [42, 86]. Glucose >200 mg/dL on CGM predicts CFRD progression, while insulin treatment targeting CGM fluctuations has been associated with improved pulmonary function and weight gain [87–89].

**Key Practice Points—Screening and Diagnosis of CFRD:**

• Annual CFRD screening is recommended for all PwCF ≥10 years, with emerging evidence supporting earlier screening—particularly in children with IGT or INDET.
• Screening should occur during clinical stability, preferably with OGTT or CGM, and is especially indicated when pulmonary function or nutritional status declines.
• OGTT remains the gold standard for CFRD diagnosis due to superior sensitivity and its association with clinical deterioration; fasting glucose and HbA1c are less reliable in CF.
• Abnormal OGTT results (IGT/INDET) do not mandate treatment but require closer monitoring, as progression to CFRD is common and OGTT patterns may fluctuate overtime.
• CFRD is diagnosed at the first meeting of diagnostic criteria, even if later OGTT values normalize.
• CGM is valuable for glucose pattern assessment and treatment monitoring, but lacks validated diagnostic criteria for screening or diagnosing CFRD.
• CGM‐detected glucose >200 mg/dL predicts CFRD progression, and insulin treatment guided by CGM may improve pulmonary function and weight.

## 6. Treatment

### 6.1. Insulin Treatment

#### 6.1.1. Indications of Insulin Treatment

*Established CFRD:* Insulin insufficiency is the hallmark of CFRD, making insulin replacement the preferred therapeutic treatment, regardless of the presence of FH. Insulin is recommended as first‐line treatment in PwCFRD due to its proven benefits in improving glycemic control, pulmonary function, nutritional status, linear growth in children, and overall survival. Evaluating insulin efficacy in PwCF is challenging and generally requires multicenter collaboration. The landmark CFRD Therapy (CFRDT) Trial in 2009, conducted across 14 centers in North America and the United Kingdom over 6 years, transformed global clinical practice by establishing insulin as the standard treatment for PwCFRD without FH [40]. This trial aimed to evaluate the impact of diabetes treatment on BMI, as micro and macrovascular complications are uncommon in PwCFRD without FH, and the need for treatment in this group has been uncertain. This three‐arm, multicenter RCT compared the effects of 1‐year preprandial insulin aspart, repaglinide, or oral placebo in PwCF having abnormal glucose tolerance (61 CFRD without FH and 20 severe IGT). In the CFRD without FH group (preprandial insulin aspart, *n* = 23; repaglinide, *n* = 22; and oral placebo, *n* = 16), all subgroups showed BMI decline in the year prior to study enrollment, but insulin effectively reversed this trend, increasing BMI from −0.30 ± 0.21 kg/m^2^ to 0.39 ± 0.21 kg/m^2^ after 1 year (*p* = 0.02), while repaglinide produced only a transient 6‐month increase in BMI that was not sustained at 12 months, and no significant changes were observed in the placebo group [40]. Other studies with 1–3 years of follow‐up similarly reported improvements in BMI and pulmonary function (FEV_1_), alongside a reduction in the incidence of acute pulmonary infections following insulin initiation [3, 90, 91]. Early initiation of insulin has been linked to a progressive decline in CFRD‐related mortality, helping to narrow survival disparities between PwCF with and without diabetes and between genders [5].

Despite these benefits, some studies find no clear differences between treatment options. The most comprehensive assessment to date is the Cochrane systematic review by Onady et al. [92] in 2020, which identified 29 trials (46 references) but included only four RCTs comprising 145 participants in the final analysis. The details of these trials are given in Table 2 [40, 93–95]. Across these studies, no pharmacologic treatment demonstrated clear superiority in improving glycemic control, pulmonary function, nutritional status, or quality of life, and mortality outcomes were not reported. The overall quality of evidence was rated as “low” to “very low,” primarily due to small sample sizes, methodological limitations, and heterogeneity in study designs. Moreover, the limited number of RCTs further undermines the reliability of the conclusions. The review concluded that current evidence does not support the superiority of any pharmacologic agent (e.g., insulin or repaglinide) in the management of CFRD.

**Table 2:** Summary of randomized control trials from the cochrane review [92].

Author, year, reference	Study design and duration	Population	Intervention (s)	Key findings
Moran et al., 2001 [93]	Short term (2‐month), single‐center, crossover trial	7 adult PwCFRD without FH	Preprandial insulin lispro vs. repaglinide vs. no treatment	• Insulin lispro significantly reduced peak glucose levels and 2‐/5‐h glucose AUC values and increased 5‐h insulin AUC. • Repaglinide reduced only 5‐h glucose AUC • Neither normalized glucose excursions → dosing likely suboptimal

Moran et al., 2009 [40]	CFRDT Trial, 1 year, three arm, multicenter (14 centers in North America and the United Kingdom) RCT	61 PwCFRD without FH + 20 severe IGT	Preprandial insulin aspart vs. repaglinide vs. Placebo	• In the CFRD without FH group (preprandial insulin aspart, *n* = 23; repaglinide, *n* = 22; and oral placebo, *n* = 16), all subgroups showed a decline in BMI during the 12 months preceding study enrollment. • Only insulin reversed chronic weight loss after 1 year of treatment (BMI: −0.30 ± 0.21 kg/m^2^ → + 0.39 ± 0.21 kg/m^2^, *p* = 0.02). • Repaglinide: produced only a transient BMI increase during the first 6 months that was not sustained at 12 months • Placebo: no siginificant BMI change • No significant differences observed among groups in terms of pulmonary function (annual FEV_1_ or FVC decline or frequency of acute exacerbations), HbA1c, or fasting glucose levels, although postprandial glucose was lower with insulin. • The authors noted that this was expected, as prior evidence indicates that at least 4 years of follow‐up are needed to detect pulmonary function differences in PwCF across glucose tolerance categories [10]. • However, Onady et al. [92] interpreted these results more critically, arguing that none of the three treatment groups (insulin, repaglinide, placebo) showed superiority over the others.

Ballmann et al., 2018 [94]	2‐year, multicenter, open‐label RCT	PwCFRD (Insulin *n* = 37; Repaglinide *n* = 30)	Insulin vs. repaglinide	• No differences in HbA1c, pulmonary function, or adverse events between groups. • BMI z‐score improved at 12 months with insulin but not sustained at 24 months. • Results limited by high dropout, variable insulin dosing between centers, and small sample size. The lack of improvement in BMI in either group—contrasting with earlier studies reporting insulin‐associated weight gain in CFRD.

Grover et al., 2008 [95]	12‐week, crossover RCT	19 adult PwCFRD without FH	Bedtime glargine vs. NPH insulin	• Glargine achieved a greater reduction in fasting plasma glucose (*p* = 0.03) and showed a trend toward weight gain (*p* = 0.07) without severe hypoglycemia. • No differences in HbA1c or postprandial glucose between groups • At study completion, all participants chose to continue glargine afterward

Abbreviations: AUC, area under curve, CFRD, cystic fibrosis related diabetes; FEV1, forced expiratory volume in 1 s; FH, fasting hyperglycemia; FVC, forced vital capacity; IGT, impaired glucose tolerance; NPH, neutral protamine Hagedorn; PwCF, patients with CF; PwCFRD, patients with CFRD; RCT, randomized controlled trial.

At present, due to its anabolic effects, well‐established safety profile, and consistent clinical benefits, insulin remains the standard recommended treatment in PwCFRD. However, larger, rigorously designed, long‐term RCTs are needed to better define the optimal therapeutic approach and its impact on clinical outcomes.

#### 6.1.2. Early Glucose Abnormalities (INDET or IGT)

PwCF may develop impaired insulin secretion and hyperglycemia before meeting the diagnostic criteria for CFRD during an OGTT. For individuals whose OGTT results fall within the prediabetic range, existing data do not justify the regular use of insulin treatment. Although insulin has potential benefits, such as anabolic effects, it also increases the overall treatment burden of CF and carries a risk of hypoglycemia. In individuals with INDET or IGT, treatment burden plays a significant role in deciding whether to initiate insulin and in selecting the most appropriate regimen.

Despite the lack of evidence for routine use, some centers and clinical guidelines recommend early initiation of insulin in selected prediabetic PwCF who show signs of worsening nutritional status, deteriorating pulmonary function, or hyperglycemic symptoms such as polydipsia or polyuria [89, 96, 97]. Several nonrandomized observational studies support this approach, reporting improvements in weight and pulmonary function with early insulin intervention in individuals with early glucose abnormalities who do not yet meet OGTT criteria for CFRD (Table 3) [90, 97–102].

**Table 3:** Summary of studies suggesting insulin treatment improves pulmonary function and nutritional status in patients with cystic fibrosis with early glucose abnormalities.

Author, year, reference	Study/design	Population	Insulin regimen	Key findings
Dobson et al., 2002 [98]	Random BGL elevation despite normal OGTT (observational)	Four PwCF with random BGL 200–325 mg/dL, normal OGTT	Low‐dose insulin (0.12–0.18 U/kg/day)	Improved pulmonary function and weight gain within 3 months
Franzese et al., 2005 [99]	Basal insulin in chronic and intermittent CFRD (observational)	Four chronic CFRD aged 15–29 years (previously treated with prandial bolus insulin for 1–3 years) vs. four intermittent CFRD aged 10–21 years (who had required insulin only during pulmonary exacerbation) vs. six intermittent CFRD controls aged 14–18 years (who did not receive insulin)	Glargine 0.3 U/kg/day	After 6 months: pulmonary infections decreased in both chronic group (3.75 ± 0.5 →1.75 ± 0.9, *p* < 0.01) and intermittent group (2.75 ± 0.50 →1.25 ± 0.5, *p* < 0.001); BMI and HbA1c stayed stable in groups; no hypoglycemic event reported. Authors suggested that basal insulin may reduce pulmonary infections in both diabetic and prediabetic PwCF without increasing hypoglycemia risk; however, the short study duration may have limited the ability to detect changes in BMI, FEV1, and HbA1c.
Mozzillo et al., 2009 [90]	Basal insulin in dysglycemia (prospective)	22 children (nine CFRD, nine IGT, four with abnormal CGM results but normal OGTT)	Glargine 0.2 U/kg/day	Over 12 months: 8.8% increase in FEV_1_; ~50% reduction in pulmonary exacerbations, improvement in BMI Z‐score in those with baseline values <–1.
Hameed et al., 2012 [100]	Australian pediatric pilot study conducted by the group that later led the CF‐IDEA trial (prospective)	18 children (12 INDET/early insulin deficiency, six CFRD), aged 7.2–18.1 years	Basal insulin detemir (initiated at 0.1 U/kg/day and titrated to 0.13 U/kg/day)	After median treatment duration of 0.8 years: weight loss reversed, FEV_1_ increased by 3.7 ± 10.6%, with greater improvement (5.3 ± 11.5%) observed in those with early insulin deficiency.

Abbreviations: BGL, blood glucose levels; CFRD, cystic fibrosis related diabetes; CGM, continous glucose monitoring; CF‐IDEA trial, cystic fibrosis–insulin deficiency, early action trial; FEV_1_, forced expiratory volume in 1 s; FH, fasting hyperglycemia; INDET, indeterminate glycemia; OGTT, oral glucose tolerance test, PwCF, Patients with CF.

The CF‐IDEA trial provided important new insights into early insulin intervention in PwCF with early glycemic abnormalities [103]. Until the recently completed CF‐IDEA trial, only two small randomized studies had evaluated insulin treatment in patients with early glycemic abnormalities but without CFRD, and neither demonstrated a significant benefit [40, 104]. The CFRDT trial primarily included PwCFRD but also enrolled 20 participants without diabetes who exhibited severe IGT on OGTT (120‐min glucose: 180–200 mg/dL). Of these participants, seven were randomly designated to receive insulin, four were allocated to repaglinide, and nine to an oral placebo, with neither the insulin nor repaglinide groups demonstrating any improvement in BMI, pulmonary function, or hospitalization rates [40]. Similarly, Minicucci et al. [104] randomized participants without CFRD but with declining BMI and pulmonary function and 120‐min OGTT glucose levels of 140–200 mg/dL, to once‐daily insulin glargine (*n* = 18) or observation only (*n* = 16). The groups did not differ significantly with respect to BMI, HbA1C or FEV_1_, except for a modest improvement in HbA1C observed in the glargine arm at 18 months (*p* = 0.04) [104].

The CF‐IDEA trial, the first adequately powered multicenter RCT to evaluate the necessity of insulin treatment in PwCF with early glycemic abnormalities prior to the onset of CFRD, was conducted across five pediatric hospitals in Australia and one in the United States, enrolling children aged 5–18 years with CF who exhibited abnormal glucose tolerance but had not yet developed CFRD [103]. Participants were classified according to OGTT peak glucose levels: 148–200 mg/dL as CF insulin deficiency Stage 1 and ≥200 mg/dL as Stage 2, and were randomly assigned to either once‐daily long‐acting insulin detemir (starting at 0.1 U/kg/day with 0.5 U titrations to maintain finger‐stick glucose 72–144 mg/dL) or observation. The main endpoints included changes in weight Z‐score, percent‐predicted FEV_1_ (ppFEV_1_), and percent‐predicted FVC (ppFVC), while safety assessments covered hypoglycemia, insulin‐associated side effects, and CGM‐derived time in hypoglycemia. Of 109 enrolled participants (55 insulin, 54 observation), 104 completed the 12‐month study. No statistically or clinically meaningful improvements were observed in pulmonary function, nutritional status, or glycemic control between the insulin and observation groups, nor between stages 1 and 2, and no severe hypoglycemia or insulin‐related adverse effects reported. These findings indicate that routine early insulin initiation before meeting diagnostic OGTT criteria for CFRD is not supported by current evidence and should be reserved for individualized cases or research settings. The CF‐IDEA trial underscores the importance of regular glucose monitoring and timely diagnosis of CFRD rather than preemptive pharmacologic intervention as the optimal strategy to preserve metabolic and clinical health in this population. One of the strengths of the CF‐IDEA trial was that statistical analyses were performed not only within the insulin group but also between the randomized groups, thereby avoiding regression‐to‐the‐mean bias in the main study endpoints [105]. The study was powered, based on previously published pilot data, to detect differences in weight Z‐score and pulmonary function between the insulin and observation groups [41, 100]. Sample size calculations were validated after 68% of participants had finished the study protocol, and the final enrollment modestly surpassed the target to compensate for the few individuals who discontinued participation after the first 6 months of the 1‐year trial. The declines in weight and pulmonary function observed prior to insulin initiation in the previous pilot study, where PwCFRD stage 1 or 2 showed marked decreases in weight Z‐score (–0.35) and pulmonary function (ppFEV_1_ –9.0; ppFVC –5.9) during the 12 months preceding insulin treatment, were not seen in the observation group of the CF‐IDEA trial. Similarly, in the CFRDT trial participants showed declines in ppFEV_1_ (–5.7) and ppFVC (–5.8) during the 12 months before randomization, whereas CF‐IDEA participants exhibited only minimal reductions in the year prior to enrollment (weight Z‐score –0.06; ppFEV_1_ –1.4; ppFVC –1.4), with these parameters remaining stable in the observation group throughout 1‐year follow‐up—an outcome that may reflect the overall improvements in CF care achieved in recent years [106].

More recently, a randomized study investigating the effects of exogenous insulin on protein catabolism in clinically stable youth with CF and early dysglycemia (IGT or INDET) demonstrated that baseline protein catabolism was surprisingly within the normal range, indicating better overall health and more efficient protein turnover than seen in earlier cohorts [43]. Authors concluded that premeal or basal insulin treatment does not confer an anabolic benefit in clinically well PwCF with abnormal glucose tolerance and is therefore not indicated prior to CFRD onset, implying that the benefit of early insulin treatment may be limited in contemporary, clinically stable populations. CFTR modulators—one of the most significant therapeutic advances in CF care—likely contribute to these findings by mitigating declines in weight and pulmonary function, although some mutations remain unresponsive to this therapy [107].

#### 6.1.3. General Principles of Insulin Treatment

Insufficient endogenous insulin production is the key abnormality in CFRD, making insulin the treatment of choice; however, dosing, regimen, and glucose targets differ from those established for T1D and T2D [79]. In CFRD, insulin should be administered at the highest safe dose to mitigate the adverse metabolic effects of insulin deficiency while maintaining HbA1c at the lowest feasible level. Optimal glycemic control minimizes hyperglycemia, prevents hypoglycemia, and improves nutritional status, pulmonary function, and overall clinical outcomes. Principles of insulin treatment are given in Table 4.

**Table 4:** Principles of insulin treatment in CFRD.

General principles	• Compared to individuals with T1D, PwCFRD generally require lower total daily insulin doses (0.5–0.8 U/kg) in normal healthy condition with a greater proportion coming from bolus insulin [108, 109]. • Higher doses may be necessary in certain stress‐related conditions such as acute pulmonary exacerbations or systemic glucocorticoid usage [72]. • A study of 18 adolescents and 137 adults with CFRD on insulin treatment found similar, modest insulin requirements (<0.5 units/kg/day) across age groups, suggesting preserved endogenous insulin secretion sufficient to compensate for pubertal insulin resistance in adolescents with CF [110]. • Insulin doses should be optimized to prevent catabolism by administering the highest amount the patient can safely tolerate without causing hypoglycemia [72]. • Insulin regimens must be individualized to meet each patient’s specific needs by selecting the approach that best aligns with their lifestyle and daily demands. • Early CFRD can often be managed with a single daily basal insulin injection to reach glycemic goals, offering lower treatment burden than multiple injections. • In PwCFRD without FH, treatment options include premeal bolus insulin, basal insulin, or a combination, depending on dietary patterns and glycemic profiles. • If fasting hypoglycemia occurs with persistent postprandial hyperglycemia on single‐dose basal insulin, reduce basal and add prandial bolus insulin before meals. • In cases of marked dysglycemia, combined basal–bolus therapy may be needed.

Basal insulin	• Long‐acting insulin analogs (e.g., detemir and glargine) are recommended, started in the early morning at 0.1 U/kg and titrated in 0.1 U/kg increments to a target of 0.25 U/kg/day. • The goal is to keep postprandial BGLs <140 mg/dL while minimizing hypoglycemia. • If fasting hypoglycemia before lunch (BGL <70 mg/dL) occurs, the basal dose may be split—two‐thirds in the morning, one‐third in the evening—with further adjustment based on glucose monitoring.

Prandial insulin (meal coverage)	• Because postprandial hyperglycemia is often predominant with relatively normal fasting BGLs, prandial rapid‐acting insulin analogs alone—without basal insulin—may be a suitable treatment option in some patients [111]. • Recommended starting prandial dose (rapid‐acting insulin): 0.5–1 U per 15 g carbohydrate, increasing by 0.5 U/15 g to reach target 2‐h postprandial levels. • In children, insulin dosing should be individualized based on age, weight, and pubertal status, as children with CFRD are generally more insulin‐sensitive than those with T1D, with insulin resistance usually emerging later, often during infections or corticosteroid use. • Suggested pediatric starting dose: 0.05–0.1 U/kg per meal, with adjustments for age, weight, and puberty [77].

Correction dose	• Correction doses can be used to manage hyperglycemia, with dosing iniated at 0.5–1 U of rapid‐acting insulin per 50 mg/dL increase in glucose levels above 150 mg/dL, adjusted according to glucose monitoring.

Overnight enteral drip feeding	• Insulin management should be adjusted based on feeding duration, ensuring optimal glycemic control. • For 8‐h feeds: combination of a regular (or rapid‐acting) insulin for initial phase and intermediate‐acting insulin, such as neutral protamine hagedorn (NPH), for later phase. • For 12‐h feeds: Insulin detemir alone • Iniating dose: calculate total carbohydrate intake, determine insulin dose adjusted for insulin‐to‐carbohydrate ratio (0.5–1.0 U/15 g), and administer half as regular insulin and half as NPH for 8‐h feeds or only as detemir for 12‐h feeds. • BGLs measured at 4‐h and the end of feeding (8‐h) are used to adjust the regular insulin and NPH doses, respectively. • Detemir insulin dose is modified based on BGLs at the end of feeding (12‐h) • If hyperglycemia is present at the onset of feeding a small dose of a rapid‐acting insulin analog can be given to help correct the elevated glucose levels.

Hospitalized patients with pulmonary exacerbations or those receiving glucocorticoids	• Basal insulin doses may be increased up to 0.2 U/kg • Add prandial bolus insulin during acute infections • Insulin needs may further increase with corticosteroid administration

Inability to achieve glycemic targets with the aforementioned regimens, and/or a preference for more physiological insulin replacement	Continuous subcutaneous insulin infusion (CSII) pumps

*Note*: Modified from: Ode KL, Ballman M, Battezzati A, Brennan A, Chan CL, Hameed S, Ismail HM, Kelly A, Moran AM, Rabasa‐Lhoret R, Saxby NA, Craig ME. ISPAD Clinical Practice Consensus Guidelines 2022: Management of cystic fibrosis‐related diabetes in children and adolescents. Pediatr Diabetes. 2022;23(8):1212−1228.

Abbreviations: BGLs, blood glucose levels; CFRD, cystic fibrosis related diabetes; NPH, neutral protamine hagedorn insulin; PwCFRD, patients with cystic fibrosis related diabetes.

In managing PwCFRD on insulin treatment, hypoglycemia (BGL <70 mg/dL) generally requires dose adjustments, with basal insulin reduction for fasting lows and prandial insulin modification for postprandial hypoglycemia. Persistent hyperglycemia may necessitate increased basal insulin, additional bolus doses, or timing adjustments with meals. Individualized glucose monitoring, incorporating CGM where available, enhances insulin titration, optimizes glycemic control, and improves pulmonary function in PwCFRD.

#### 6.1.4. Glycemic Targets and Glucose Monitoring During Insulin Treatment

Although HbA1c is not advised for use in diagnosing CFRD because of its limited sensitivity for detecting early glucose abnormalities, it remains valuable for monitoring long‐term glycemic trends and assessing the efficacy of insulin treatment, with quarterly testing recommended in insulin‐treated patients [71, 72]. An HbA1c target of ≤7% is advised to minimize the risk of microvascular complications, though individualization is essential, as some patients may require higher or lower targets based on their clinical condition [112]. Stricter goals (<5.5%) may be beneficial for selected patients by enhancing pulmonary function and reducing the number of acute pulmonary exacerbations, but their overall impact on CFRD outcomes remains unclear. PwCFRD often present with HbA1c levels <6% or in the marginal dysglycemic range (6%–6.5%) due to the shortened red blood cell lifespan in chronic diseases. An increase in HbA1c during follow‐up in an insulin‐treated PwCFRD may indicate insufficient insulin treatment.

For safe and effective glycemic control, PwCFRD on insulin should monitor BGLs via SMBG (self‐monitoring of blood glucose) no fewer than three times per day, with measurements ideally including fasting levels, or alternatively use CGM to support achievement of glycemic targets and guide insulin dose adjustments [71, 72]. Preprandial BGL monitoring is crucial, not only for insulin dose adjustments during stable periods and acute illness, but also because PwCF may experience spontaneous hypoglycemia even in the absence of diabetes or insulin treatment [56]. Ideally, both fasting and postprandial BGLs should be monitored—after each meal and at bedtime—in patients on multiple daily injection (MDI) regimens. Target values are 70–130 mg/dL for fasting, <140 mg/dL at 1 h postprandial, and <180 mg/dL at 2 h [71, 79]. If basal insulin is used, overnight glucose monitoring at midnight and/or 3 am is recommended, particularly during dose adjustments. While it is typically advised to monitor glucose levels 2‐h postmeal, recent discussions suggest that ideal timing for checking BGLs may be 1‐h after meals to optimize pulmonary function benefits from insulin treatment. This new proposal comes after the study found that 1‐h OGTT glucose, not 2‐h stimulated glucose, was adversely related with pulmonary function in PwCF [113].

With newer CGM devices, real‐time glucose readings can replace SMBG. CGM provides real‐time glucose variability, time‐in‐range, and nocturnal glycemic trends, facilitating insulin dose adjustments, preventing extreme glucose fluctuations, and potentially improving pulmonary function [114]. Fingerstick glucose measurements remain essential for validating CGM data. CGM is particularly beneficial for PwCFRD who do not respond adequately to conventional treatments, particularly those with poor growth despite insulin treatment. ISPAD suggests that CGM targets in CFRD may align with those for other diabetes types [79]. For PwCFRD, the recommended time in range target is ≥70% of glucose readings between 70–180 mg/dL, while time below range should be <4% for values below 70 mg/dL and <1% below 54 mg/dL to minimize hypoglycemia risk. Time above range should be <25% above 180 mg/dL and <5% above 250 mg/dL to reduce hyperglycemia‐related complications [115]. These targets balance glycemic control and pulmonary health benefits while mitigating the risk of hypoglycemia.

#### 6.1.5. Insulin Doses and Types of Insulin Regimen

There is considerable variability in insulin regimen selection and initial doses for PwCFRD, influenced by an individual’s physiological, psychological, and social factors, including the capability to administer MDIs, dietary habits (e.g., frequent snacking), use of systemic corticosteroids, the predominant pattern of hyperglycemia (postprandial vs. fasting), physical activity levels, history of hypoglycemia, or impaired hypoglycemia awareness. Access to and the affordability of insulin and diabetes technologies—such as CGM and continuous subcutaneous insulin infusion (CSII)—also substantially influence treatment decision‐making. The details of insulin dosing and regimens used are provided in Table 4.

#### 6.1.6. Continuous Subcutaneous Insulin Infusion (CSII) Pumps

As an alternative to MDIs, CSII pumps exclusively utilizes rapid‐acting insulin, allowing for flexible adjustments based on snacks, meals, and nocturnal feeds. The pump delivers insulin at a programed basal rate (units/hour), with customizable dose modifications throughout the day. They function in open loop (manual glucose entry), partial closed‐loop (algorithm‐driven insulin adjustments), or hybrid closed‐loop (automated insulin delivery with minimal user input) systems. Insulin‐to‐carbohydrate ratios follow standard calculations, and regular blood glucose monitoring remains essential, particularly before and after meals and at bedtime. While extensively studied in T1D, emerging data suggest potential benefits for CFRD management. Small studies indicate that insulin pump usage without CGM improves glycemic control and lean body mass in CFRD, primarily by enhancing meal and snack coverage [116]. Insulin pumps optimize carbohydrate‐insulin matching, particularly during overnight enteral feedings. Square‐wave and dual‐wave boluses, as well as increased basal rates, help manage nocturnal glucose fluctuations.

The integration of CGM further enhances insulin pump efficacy in CFRD management. Hybrid closed‐loop systems automatically calibrate insulin delivery based on CGM data, preventing hypoglycemia and correcting hyperglycemia [117, 118]. Transitioning from an open loop system with CGM to a partial closed‐loop system has been linked to increased time in target glucose range without added hypoglycemia risk [119]. A pilot study of closed‐loop systems in CFRD showed no significant change in mean glucose but demonstrated improved treatment satisfaction and reduced burden [117]. However, long‐term studies are needed to assess their effectiveness in PwCF compared to those with T1D. CSII offers theoretical benefits in CFRD, such as improved flexibility and reduced hypoglycemia risk, but remains underutilized—possibly due to cost or treatment burden in an already complex disease.

### 6.2. Nutritional Management

Nutritional management in CFRD differs significantly from other types of diabetes due to its unique metabolic demands and low cardiovascular risk. Unlike T1D or T2D, where intake of calories, fat, protein, and sodium is often restricted, PwCFRD requires higher nutritional intake. Most PwCF rely on strategies to boost calorie consumption, including the use of calorie‐rich beverages, to meet their elevated energy needs. A high‐calorie, high‐fat, and high‐salt diet is essential, with caloric intake generally ≥120%–150% of estimated energy needs to maintain a BMI at or above the 50th percentile for age and sex. Fat should account for at least 40% of total calories, carbohydrates for 45%–50%, and protein intake should be increased to twice the recommended daily allowance for age, even in those with kidney disease. Salt should not be restricted due to increased losses. Caloric restriction is generally not recommended in PwCF, except in older patients with less severe variants who are classified as overweight or obese. Consumption of sugar‐sweetened beverages is generally avoided, particularly in PwCFRD with poor glycemic control. In PwCFRD who struggle with glycemic control despite optimized insulin treatment, shifting toward lower‐glycemic carbohydrate sources and evenly distributing carbohydrate intake throughout the day may help improve glucose stability without reducing overall caloric intake [71, 120, 121]. Additionally, CFTR modulator therapy can lead to significant weight gain, further emphasizing the need for individualized nutritional strategies.

### 6.3. Other Treatment Options

#### 6.3.1. Oral Hypoglycemic Agents

Oral hypoglycemic agents are generally not advised for managing CFRD due to limited efficacy and insufficient evidence. Their ability to stimulate insulin production is compromised by the reduced *β*‐cell mass in PwCF [79]. Clinical trials, including randomized studies, have shown that while agents like repaglinide—an agent that stimulates insulin production from *β*‐cells—may lead to short‐term benefits such as a temporary increase in BMI during the first 6 months of treatment, this effect was not sustained, as there was no significant difference in BMI change after 1 year compared to the year before treatment [40]. A 2‐year multicenter randomized trial comparing repaglinide and insulin found no significant differences in HbA1c, BMI, pulmonary function, or side effects, leading authors to conclude that repaglinide is as effective and safe as insulin for maintaining glycemic control [94]. However, in both studies the insulin dose was either not documented or highly variable, raising the possibility that results in the insulin‐treated groups were negatively influenced by insufficient dosing [122]. According to the ISPAD guidelines, in specific circumstances—particularly when asymptomatic PwCFRD without FH detected through annual screening refuse insulin treatment—a closely supervised trial of oral anti‐diabetic medications may be considered [79].

Further concerns of non‐insulin treatments include the potential for insulin secretagogues to accelerate *β*‐cell decline and the limited relevance of insulin sensitizers, given that insulin resistance is not a primary feature of CFRD. Additionally, the side effects of insulin sensitizers make them less suitable for PwCF—metformin commonly causes gastrointestinal disturbances, while thiazolidinediones increase the risk of osteoporosis—both of which are prevalent concerns in CF.

Incretin‐based therapies, including glucagon‐like peptide‐1 (GLP‐1) agonists and dipeptidyl peptidase‐4 (DPP‐4) inhibitors, are under investigation, but early studies have shown mixed results. A recent trial of sitagliptin in pancreatic‐insufficient PwCF showed no significant improvement in glucose or insulin response. Thus, insulin remains the standard of care, and more robust research is needed before recommending non‐insulin therapies for CFRD.

The effectiveness of incretin hormones—GLP‐1 and glucose‐dependent insulinotropic polypeptide (GIP)—in stimulating insulin secretion in PwCF remains uncertain. Several RCTs have investigated this, including a recent double‐blind study of 32 PwCF with IGT, which found that GLP‐1, but not GIP, stimulated glucose‐dependent insulin secretion [123]. Other studies involving incretin mimetic agents, including GLP‐1 agonists and DPP‐4 inhibitors, demonstrate conflicting results. In a small cohort of six PwCF with IGT, exenatide (a GLP‐1 agonist) reduced postprandial hyperglycemia by slowing gastric emptying rather than increasing insulin secretion [124]. Similarly, a study on sitagliptin (a DPP‐4 inhibitor) found improved meal‐related incretin responses, early insulin release, and glucagon suppression, but no effect on postprandial glucose, overall glucose tolerance, second‐phase insulin response, or *β*‐cell sensitivity [125]. These conflicting findings highlight the need for further research before incretin‐based therapies can be recommended in CFRD management.

**Key Practice Points—Treatment of CFRD**:

• Insulin is the first‐line treatment for all individuals with established CFRD, regardless of fasting hyperglycemia, due to its proven benefits for BMI, pulmonary function, nutritional status, growth, and survival.
• Evidence for non‐insulin agents is limited; current trials show no clear superiority of any pharmacologic alternative, and overall evidence quality remains low.
• Routine insulin treatment is not recommended for early glucose abnormalities (IGT or INDET).
• The CF‐IDEA trial demonstrated no clinical benefit of insulin in children with early dysglycemia, supporting a focus on monitoring rather than preemptive treatment.
• Insulin treatment should be individualized, using the highest safe dose to control hyperglycemia while avoiding hypoglycemia.
• Quarterly HbA1c monitoring is recommended for those on insulin; SMBG ≥3 times/day or CGM should guide titration and support glycemic targets.
• Target glucose ranges for PwCFRD: fasting 70–130 mg/dL; <140 mg/dL at 1 h postprandial; <180 mg/dL at 2 h; CGM time‐in‐range ≥70% (70–180 mg/dL).
• Insulin regimen selection (basal‐bolus vs. premix vs. pump) depends on hyperglycemia pattern, lifestyle, treatment burden, steroid use, and access to diabetes technology.
• Insulin pump therapy (CSII), especially with CGM and hybrid closed‐loop systems, may improve glycemic control and treatment flexibility, though evidence in CFRD remains limited and long‐term studies are needed.

#### 6.3.2. CFTR Modulator Therapies

The introduction of CFTR modulator therapies has markedly improved quality of life in PwCF, offering near‐curative benefits for many. These therapies restore the function of the defective CFTR protein through different mechanisms: enhancing channel gating—for example, IVA for G551D and other gating mutations—or correcting protein misfolding and trafficking, as achieved with combination therapies such as lumacaftor/IVA (LUM/IVA), tezacaftor/IVA (TEZ/IVA), and elexacaftor/tezacaftor/IVA (ELX/TEZ/IVA) used for F508del mutations. Each therapy targets specific CFTR defects to improve overall channel function [126]. CFTR modulators, particularly the triple combination ELX/TEZ/IVA, have significantly improved pulmonary outcomes; however, its effects on extrapulmonary manifestations—especially glucose metabolism and CFRD—remain an area of ongoing investigation.

CFRD is more prevalent in individuals with *CFTR* variants causing severe dysfunction, suggesting a possible direct involvement of CFTR in pancreatic islet function and raising hopes that CFTR modulators could prevent or reverse CFRD. These therapies may affect glucose tolerance and insulin dynamics by reducing inflammation, improving islet function, and enhancing incretin secretion, while improved nutrient absorption and weight gain may increase insulin resistance [66]. Although *CFTR* RNA is detected in a small subset of human islet *β*‐cells, studies have not confirmed the presence of CFTR protein in insulin‐, glucagon‐, or somatostatin‐producing cells, and CFTR modulators show no direct effect on insulin secretion in vitro [20]. Observed effects, such as reduced insulin secretion in CFTR‐deficient models, may reflect nonspecific inhibition of chloride channels in the islets rather than a direct role of CFTR [127, 128].

While preliminary evidence suggests potential metabolic benefits of CFTR modulators, they do not produce immediate improvements in CFRD or reverse established disease, and their long‐term effects on glucose metabolism remain uncertain [66, 129]. Short‐term studies show modest increases in insulin secretion and reduced insulin requirements—particularly with IVA—yet improvements in glucose tolerance remain inconsistent [66, 119]. A registry study reported a slower increase in CFRD prevalence with IVA, possibly influenced by genotype severity [130], while a clinical trial found enhanced insulin production and *β*‐cell function with reduced need for exogenous insulin but no significant improvement in glucose tolerance [131]. Evidence for LUM/IVA and ELX/TEZ/IVA is limited and mixed, with some studies showing improved glycemia or insulin sensitivity and others reporting no significant effect [132, 133]. ELX/TEZ/IVA appears most promising, showing more consistent metabolic improvements—particularly in individuals with IGT—with some patients achieving NGT after treatment and observational data further indicate reductions in CGM‐derived hyperglycemia and glycemic fluctuation without significant effects on hypoglycemia, although individual responses vary [119, 134]. Additional studies are needed to clarify these effects.

Despite promising early findings, the long‐term effects of CFTR modulators on CFRD risk and progression remain unclear due to limited and conflicting data. It is uncertain whether improved nutrition and increased lifespan will alter the CFRD phenotype or contribute to T2D risk, and associated weight gain may necessitate reassessment of caloric needs and individualized nutritional counseling. Standard CFRD screening and monitoring should continue for all PwCF on CFTR modulators, regardless of metabolic response, and close glycemic monitoring is recommended after modulator therapy initiation—especially in those with CFRD—to guide insulin adjustments and detect hypoglycemia [111]. Patients on antihyperglycemic treatment should increase glucose monitoring and obtain HbA1c every 3 months during the first year, although OGTT is not recommended for assessing diabetes remission [111]. Possible interactions between medications should also be taken into account, particularly for drugs metabolized through pathways shared with CFTR modulators (e.g., sulfonylureas and statins) [111].

While CFTR modulator therapies hold potential to improve glycemic outcomes in PwCF—particularly in those with IGT or newly diagnosed CFRD—further investigation is required to clarify whether early initiation of CFTR modulators can prevent or delay CFRD onset. Large, multicenter pediatric studies are essential to evaluate the long‐term clinical, metabolic, and developmental effects of these therapies. While short‐term metabolic benefits are increasingly evident, the long‐term impact on both pulmonary and extrapulmonary outcomes, including glucose regulation, remains uncertain and warrants further investigation.

**Key Practice Points—CFTR Modulator Therapies:**

• CFTR modulators, especially ELX/TEZ/IVA, improve pulmonary outcomes, but their effects on glucose metabolism and CFRD are variable and not fully defined.
• They may modestly enhance insulin secretion or glycemia, particularly in IGT, but do not reverse established CFRD, and long‐term metabolic effects remain uncertain.
• Improved nutrition and weight gain may increase insulin resistance, requiring individualized dietary adjustments.
• Standard CFRD screening should continue, and PwCFRD should undergo closer glucose monitoring after modulator initiation to guide insulin adjustments and detect hypoglycemia.

### 6.4. Monitoring for Complications

CFRD can lead to chronic microvascular complications, similar to other forms of diabetes, with risk influenced by the duration of diabetes and the degree of glycemic control. Notably, such complications may develop at lower HbA1c levels in CFRD compared to T1D or T2D. Therefore, PwCFRD should undergo routine monitoring comparable to that recommended for T1D (Table 5).

**Table 5:** Monitoring in patients with CFRD.

Blood glucose monitoring	Fasting blood glucose: target to 70–90 mg/dL when using basal insulin Postprandial blood glucose: target <140 mg/dL at 1–2 h

HbA1c	Check for every 3 months Target low‐normal values (<5.5%)

Continous glucose monitoring (CGM)	Screening: if a patient has dysglycemia but the insulin treatment indications are not clear. Treatment: a patient whose CFRD is difficult to manage through standard approaches such as if a patient is using insulin but has poor growth.

Screening for microvascular complications	Annual screening for microvacular complications should begin either 5 years after a CFRD diagnosis or from the time FH is first identified—whichever occurs first [71]. • Dilated eye examination for retinopathy • Spot urine albumin:creatinine ratio for nephropathy • Sensory foot examination with testing for vibration with a tuning fork and for pressure with 10 g monofilament (peripheral neuropathy)

Screening for macrovascular complications	• Blood pressure measurement at all visits • If hypertension is detected, treatment with an angiotensin‐converting enzyme (ACE) inhibitor or an angiotensin II receptor blocker should be initiated
	• Annual lipid testing is recommended only for PwCFRD with preserved pancreatic exocrine function or additional cardiovascular risk factors (post‐transplantation, obesity, or a family history of early coronary artery disease), given the low incidence of hyperlipidemia and macrovascular disease in this population [71]

Pulmonary function tests	Perform every 3 months

Abbreviations: CFRD, cystic fibrosis related diabetes; FH, fasting hyperglycemia; PwCFRD, patients with CFRD.

### 6.5. Health‐Related Quality of Life (HRQoL) in PwCFRD

The advent of CFTR modulators has transformed CF care, leading to substantial improvements in pulmonary and nutritional outcomes, as well as survival, and consequently enhancing overall HRQoL. As life expectancy increases, attention is shifting from survival to long‐term wellness. Despite better pulmonary outcomes, extrapulmonary complications—such as CFRD, chronic pain, fatigue, depression, and anxiety—now have a growing impact on HRQoL. CFRD, in particular, adds complexity to CF management by increasing treatment burden, dietary restrictions, and psychological stress—influencing emotional well‐being, daily functioning, and treatment adherence—especially as improved pulmonary health with CFTR modulators may shift the focus toward diabetes as a second chronic illness.

Studies examining the impact of CFRD on HRQoL have yielded controversial results. Some studies report no differences in HRQoL or treatment burden compared with T1D, whereas others associate worsening glycemia with an increased treatment burden [54, 135–137]. Notably, a 12‐year longitudinal study involving 234 PwCF aged 14–48 years found that CFRD adversely affected more than half of the assessed HRQoL domains, highlighting its significant impact on overall well‐being [138]. The treatment burden of CFRD is multifaceted, with insulin‐treated PwCFRD reporting significantly higher burden scores than those not on insulin—reflecting the added complexity of managing both CF and diabetes and the resulting psychological stress and lifestyle challenges—underscoring the need for large‐scale, long‐term studies to clarify these impacts and guide strategies to reduce this multidimensional burden.

In addition to clinical challenges, healthcare disparities in CFRD management are evident. Limited access to specialized CF care and screening tools such as OGTT or CGM contributes to delays in diagnosis, which are further influenced by socioeconomic status, insurance coverage, and geographic location. Treatment access also varies, with underserved communities facing greater barriers to insulin treatment, CGM, insulin pumps, and CFTR modulators. Social determinants of health—including food insecurity, limited health literacy, and restricted access to diabetes education—further affect outcomes, and racial and ethnic disparities have been associated with differences in glycemic control and CFRD complication rates [139]. Addressing these inequities requires policies that expand insurance coverage, broader use of telemedicine, and culturally tailored education and support programs to ensure more equitable CFRD care.

In clinical practice, healthcare professionals should recognize the potential adverse impacts of CFRD on overall health and provide appropriate support. This highlights the importance of comprehensive, multidisciplinary care that includes routine symptom screening to identify patients at highest risk for impaired quality of life, enabling more targeted interventions and personalized goal setting. Ongoing assessment of quality of life ensures that treatment aligns with patient‐centered outcomes, measuring success not only by clinical indicators but also by meaningful improvements in daily living. Additionally, educational programs for patients, families, and care teams remain essential to meet the evolving needs of the CF population.

## 7. Conclusion

CFRD represents a unique and increasingly important aspect of CF care as life expectancy continues to improve. While CFRD poses significant clinical challenges, it is also highly manageable with early detection, individualized insulin treatment, and continuous monitoring. The advent of CFTR modulators and the emergence of novel diabetes technologies have created new opportunities to optimize glycemic control and enhance quality of life for PwCFRD. Ongoing research evaluating the long‐term effects of CFTR modulators on insulin secretion and glycemic control, investigating noninsulin therapies, developing predictive biomarkers, and refining diagnostic tools will be instrumental in shaping future management strategies. Ultimately, integrating these advances into routine clinical care will be key to preserving pulmonary function, nutritional status, and quality of life, ensuring that PwCF can continue to thrive throughout their lifespan.

## Disclosure

The author has read and approved the final version of the manuscript.

## Conflicts of Interest

The author declares no conflicts of interest.

## Author Contributions

Dogus Vuralli conceived the study idea, designed the study, performed the literature search, conducted screening and data extraction, synthesized the data, wrote the manuscript.

## Funding

The author declares that this study received no financial support from any organization.

## Data Availability

The findings of this study are supported by data sourced from published literature, with relevant citations listed in the references section. Additionally, the data underlying the study’s results are available from the corresponding author upon reasonable request.
